# Low crude protein formulation with supplemental amino acids for its impacts on intestinal health and growth performance of growing-finishing pigs

**DOI:** 10.1186/s40104-024-01015-6

**Published:** 2024-03-25

**Authors:** Marcos Elias Duarte, Wanpuech Parnsen, Shihai Zhang, Márvio L. T. Abreu, Sung Woo Kim

**Affiliations:** Department of Animal Science, North Carolina State University, Raleigh, NC 27695 USA

**Keywords:** Amino acids, Growing-finishing pigs, Growth performance, Intestinal health, Low crude protein formulation, Net energy

## Abstract

**Background:**

Low crude protein (CP) formulations with supplemental amino acids (AA) are used to enhance intestinal health, reduce costs, minimize environmental impact, and maintain growth performance of pigs. However, extensive reduction of dietary CP can compromise growth performance due to limited synthesis of non-essential AA and limited availability of bioactive compounds from protein supplements even when AA requirements are met. Moreover, implementing a low CP formulation can increase the net energy (NE) content in feeds causing excessive fat deposition. Additional supplementation of functional AA, coupled with low CP formulation could further enhance intestinal health and glucose metabolism, improving nitrogen utilization, and growth performance. Three experiments were conducted to evaluate the effects of low CP formulations with supplemental AA on the intestinal health and growth performance of growing-finishing pigs.

**Methods:**

In Exp. 1, 90 pigs (19.7 ± 1.1 kg, 45 barrows and 45 gilts) were assigned to 3 treatments: CON (18.0% CP, supplementing Lys, Met, and Thr), LCP (16.0% CP, supplementing Lys, Met, Thr, Trp, and Val), and LCPT (16.1% CP, LCP + 0.05% SID Trp). In Exp. 2, 72 pigs (34.2 ± 4.2 kg BW) were assigned to 3 treatments: CON (17.7% CP, meeting the requirements of Lys, Met, Thr, and Trp); LCP (15.0% CP, meeting Lys, Thr, Trp, Met, Val, Ile, and Phe); and VLCP (12.8% CP, meeting Lys, Thr, Trp, Met, Val, Ile, Phe, His, and Leu). In Exp. 3, 72 pigs (54.1 ± 5.9 kg BW) were assigned to 3 treatments and fed experimental diets for 3 phases (grower 2, finishing 1, and finishing 2). Treatments were CON (18.0%, 13.8%, 12.7% CP for 3 phases; meeting Lys, Met, Thr, and Trp); LCP (13.5%, 11.4%, 10.4% CP for 3 phases; meeting Lys, Thr, Trp, Met, Val, Ile, and Phe); and LCPG (14.1%, 12.8%, 11.1% CP for 3 phases; LCP + Glu to match SID Glu with CON). All diets had 2.6 Mcal/kg NE.

**Results:**

In Exp. 1, overall, the growth performance did not differ among treatments. The LCPT increased (*P* < 0.05) Claudin-1 expression in the duodenum and jejunum. The LCP and LCPT increased (*P* < 0.05) CAT-1, 4F2hc, and B^0^AT expressions in the jejunum. In Exp. 2, overall, the VLCP reduced (*P* < 0.05) G:F and BUN. The LCP and VLCP increased (*P* < 0.05) the backfat thickness (BFT). In Exp. 3, overall, growth performance and BFT did not differ among treatments. The LCPG reduced (*P* < 0.05) BUN, whereas increased the insulin in plasma. The LCP and LCPG reduced (*P* < 0.05) the abundance of Streptococcaceae, whereas the LCP reduced (*P* < 0.05) Erysipelotrichaceae*,* and the alpha diversity.

**Conclusions:**

When implementing low CP formulation, CP can be reduced by supplementation of Lys, Thr, Met, Trp, Val, and Ile without affecting the growth performance of growing-finishing pigs when NE is adjusted to avoid increased fat deposition. Supplementation of Trp above the requirement or supplementation of Glu in low CP formulation seems to benefit intestinal health as well as improved nitrogen utilization and glucose metabolism.

## Background

In pig production, low crude protein (CP) formulation with supplemental amino acid (AA) has been widely implemented as a strategy to reduce the feed cost, and more recently to improve intestinal health mainly by decreasing the protein fermentation by intestinal microbiota [1–3]. Moreover, the reduction in the nitrogen (N) content in the diet reduces N excretion and, consequently, reduces environmental pollution [4, 5]. Use of supplemental AA facilitates meeting the ideal AA profile and also allows reducing CP in the diet, enhance improving N utilization and growth of pigs [3, 6, 7].

Typical swine diets are supplemented with 3 to 5 crystalline AA, whereas currently at least 8 feed-grade supplemental AA including Lys, Met, Trp, Thr, Val, Ile, His, and Arg are commercially available and can be used in low CP formulation [8]. However, reducing CP by more than 4% unit may cause nitrogen deficiency for endogenous synthesis of non-essential AA (NEAA) [9], production of amines, and intestinal microbial fermentation [10]. Furthermore, N deficiency can increase the deamination of essential AA for the synthesis of other N compounds [11, 12], compromising the growth performance [8]. Therefore, supplemental AA can enhance the dietary N balance to overcome the insufficiency of NEAA as N source in the diet [13, 14].

Supplementation of certain AA has been shown functional benefits beyond the nutritional properties in pigs [15, 16]. Supplementation of Trp in swine diets has been related to improved appetite by increasing the expression of ghrelin and improved intestinal health by enhancing immune response [14, 17]. According to Wang et al. [18], Trp increases the expression of tight junction proteins in intestinal epithelial cells of pigs, consequently increasing the intestinal barrier function. Additionally, supplementation of Trp reduced stress due to an increase in hypothalamic serotonin synthesis [19, 20]. Moreover, non-specific N sources have been used to supply N needed for the endogenous synthesis of NEAA [9]. Glutamate has been used in swine diets mainly for its functional roles on intestinal health, glucose metabolism, and fat deposition [13, 21–23]. It has been reported that Glu can regulate insulin secretion enhancing glucose metabolism [24, 25]. Additionally, it has been reported that dietary Glu reduces fat deposition in adult rats [26], growing chickens [27], and growing-finishing pigs [28]. According to Hu et al. [29], dietary Glu decreased backfat deposition by increasing the expression of genes associated with lipolysis in subcutaneous adipose tissue, whereas it can increase intramuscular fat deposition by increasing the expression of genes associated with lipogenesis in the muscular tissue of growing-finishing pigs.

In the application of low CP formulation with supplemental AA meeting the requirement, using metabolizable energy (ME) as the energy system can cause increased fat deposition due to the increase in the net energy (NE) content in the diet [7, 30]. Therefore, formulating a low CP diet with supplemental AA should use the NE system should prevent the unintended increment of energy [31].

Considering the research advances in the use of supplemental AA and increased availability of supplemental AA, it was hypothesized that low CP formulation with supplemental AA can improve growth performance by enhancing intestinal health, and glucose metabolism as well as increasing AA transporter and N utilization. Furthermore, increased supplementation of Trp and Glu can enhance the dietary N balance and further enhance the benefits of low CP formulation with supplemental AA. To test the hypothesis, the objective of this study was to determine the effects of low CP formulation with up to 8 supplemental AA on growth performance, expression of AA transporters, intestinal integrity, immune and inflammatory responses, and fecal microbiota and to determine the need for functional AA in low CP diets fed to growing-finishing pigs.

## Materials and methods

The experimental procedures used in this study were reviewed and approved by the Institutional Animal Care and Use Committee at North Carolina State University.

### Animals, experimental design, and diets

Three experiments were conducted in the Swine Evaluation Station of NC State University (Clayton, NC, USA). Pens (4.0 m × 1.4 m) with solid concrete floor were equipped with a nipple drinker and a 1-hole self-feeder. Pigs were ear-tagged for individual identification and had free access to feed and water.

In Exp. 1, 90 pigs (45 barrows and 45 gilts) at 19.7 ± 1.1 kg of body weight (BW) were allotted in 3 dietary treatments using a randomized complete block design with sex and initial BW as blocks. Each treatment had 10 pens (*n* = 10; 5 pens with barrows and 5 pens with gilts) and 3 pigs were housed in a pen. Treatments were CON (18% CP supplementing with crystalline Lys, Met, and Thr), LCP (16% CP supplementing with crystalline Lys, Met, Thr, Trp, and Val), and LCPT (16.05% CP; LCP + 0.05% SID Trp). All experimental diets were supplemented with crystalline AA to meet the AA requirements on NRC [32] and were formulated based on metabolizable energy (ME) with 3.4 Mcal/kg ME. Pigs were fed the assigned dietary treatment for 28 d based on one phase. The dietary composition is summarized in Table 1.

**Table 1:** Composition of diets (Exp. 1; as-fed basis)

Item	CON^a^	LCP^b^	LCPT^c^
Feedstuff, %			
Corn (yellow)	71.40	76.92	76.87
Soybean meal (48% CP)	25.00	19.00	19.00
L-Lys HCl	0.24	0.43	0.43
DL-Met	0.04	0.09	0.09
L-Thr	0.04	0.13	0.13
L-Trp	0.00	0.02	0.07
L-Val	0.00	0.03	0.03
Dicalcium phosphate	1.05	1.20	1.20
Limestone	0.85	0.80	0.80
Vitamin premix^d^	0.03	0.03	0.03
Mineral premix^e^	0.15	0.15	0.15
Salt	0.20	0.20	0.20
Poultry fat	1.00	1.00	1.00
Calculated composition			
Dry matter, %	89.14	86.06	86.07
ME, kcal/kg	3,344	3,349	3,350
NE, kcal/kg	2,508	2,538	2,539
CP, %	18.00	16.00	16.05
SID^f^ Lys, %	0.98	0.98	0.98
SID Met + Cys, %	0.55	0.55	0.55
SID Thr, %	0.59	0.59	0.59
SID Trp, %	0.18	0.17	0.22
SID Val, %	0.71	0.64	0.64
SID Ile, %	0.64	0.54	0.54
SID Phe, %	0.77	0.66	0.66
SID His, %	0.43	0.37	0.37
SID Leu, %	1.39	1.25	1.25
Ca, %	0.66	0.66	0.66
STTD^g^ P, %	0.32	0.32	0.32

^a^Control diet (CON)

^b^Low CP diet (LCP)

^c^Low CP diet + L-Trp (LCPT)

^d^Vitamin premix: the vitamin premix provided the following per kilogram of complete diet: 6,613.8 IU of vitamin A as vitamin A acetate, 992.0 IU of vitamin D_3_, 19.8 IU of vitamin E, 2.64 mg of vitamin K as menadione sodium bisulfate, 0.03 mg of vitamin B_12_, 4.63 mg of riboflavin, 18.52 mg of D-pantothenic acid as calcium pantothenate, 24.96 mg of niacin, and 0.07 mg of biotin

^e^Mineral premix: the trace mineral premix provided the following per kilogram of complete diet: 4.0 mg of Mn as manganous oxide, 165 mg of Fe as ferrous sulfate, 165 mg of Zn as zinc sulfate, 16.5 mg of Cu as copper sulfate, 0.30 mg of I as ethylenediamine di-hydroiodide, and 0.30 mg of Se as sodium selenite

^f^*SID* Standardized ileal digestible

^g^*STTD* Standardized total tract digestible

In Exp. 2, 72 pigs (36 barrows and 36 gilts) at 34.2 ± 4.2 kg of BW were allotted to 3 dietary treatments based on a complete randomized block design using sex and initial BW as blocks. Each treatment had 8 pens (*n* = 8; 4 pens with barrows and 4 pens with gilts) and 3 pigs were housed in a pen. Treatments were CON (17.7% CP; meeting Lys, Met, Thr, and Trp); LCP (15.0% CP; meeting Lys, Thr, Trp, Met, Val, Ile, and Phe); and VLCP (12.8% CP; meeting Lys, Thr, Trp, Met, Val, Ile, Phe, His, and Leu). All experimental diets were supplemented with crystalline AA to meet the AA requirements on NRC [32] and were formulated based on ME with 3.4 Mcal/kg ME. Pigs were fed for 21 d based on one phase. The dietary composition is summarized in Table 2.

**Table 2:** Composition of diet (Exp. 2; as-fed basis)

Item	CON^a^	LCP^b^	VLCP^c^
Feedstuff, %			
Corn (yellow)	73.89	78.94	84.74
Soybean meal (48% CP)	21.21	15.62	8.90
L-Lys HCl	0.36	0.54	0.75
L-Met	0.08	0.13	0.19
L-Thr	0.10	0.18	0.28
L-Trp	0.00	0.04	0.08
L-Val	0.00	0.09	0.21
L-Ile	0.00	0.03	0.07
L-Phe	0.00	0.00	0.12
L-His	0.00	0.00	0.15
Dicalcium phosphate	1.13	1.22	1.34
Limestone	0.83	0.81	0.79
Vitamin premix^d^	0.03	0.03	0.03
Mineral premix^e^	0.15	0.15	0.15
Salt	0.22	0.22	0.22
Poultry fat	2.00	2.00	2.00
Calculated composition			
Dry matter, %	89.23	89.19	89.18
ME, kcal/kg	3,396	3,404	3,409
NE, kcal/kg	2,572	2,598	2,622
CP, %	16.69	14.81	12.59
SID^f^ Lys, %	0.98	0.98	0.98
SID Met + Cys, %	0.55	0.55	0.55
SID Thr, %	0.59	0.59	0.59
SID Trp, %	0.17	0.17	0.17
SID Val, %	0.64	0.64	0.64
SID Ile, %	0.57	0.51	0.51
SID Phe, %	0.69	0.59	0.59
SID His, %	0.39	0.34	0.34
SID Leu, %	1.29	1.16	0.99
Ca, %	0.66	0.66	0.66
STTD^g^ P, %	0.31	0.31	0.31
Analyzed composition			
CP, %	17.70	15.00	12.80

^a^Control diet (CON)

^b^Low CP diet (LCP)

^c^Very low CP diet (VLCP)

^d^Vitamin premix: the vitamin premix provided the following per kilogram of complete diet: 6,613.8 IU of vitamin A as vitamin A acetate, 992.0 IU of vitamin D_3_, 19.8 IU of vitamin E, 2.64 mg of vitamin K as menadione sodium bisulfate, 0.03 mg of vitamin B_12_, 4.63 mg of riboflavin, 18.52 mg of D-pantothenic acid as calcium pantothenate, 24.96 mg of niacin, and 0.07 mg of biotin

^e^Mineral premix: the trace mineral premix provided the following per kilogram of complete diet: 4.0 mg of Mn as manganous oxide, 165 mg of Fe as ferrous sulfate, 165 mg of Zn as zinc sulfate, 16.5 mg of Cu as copper sulfate, 0.30 mg of I as ethylenediamine di-hydroiodide, and 0.30 mg of Se as sodium selenite

^f^*SID* Standardized ileal digestible

^g^*STTD* Standardized total tract digestible

In Exp. 3, 72 pigs (36 barrows and 36 gilts) at 54.1 ± 5.4 kg of BW were allotted to 3 dietary treatments based on a complete randomized block design using sex and initial BW as blocks. Pigs used in Exp. 2 were randomly allotted in Exp. 3. Prior to the data analysis, a covariate test was conducted and there was no carryover effect from previous Exp. 2. Each treatment had 8 pens (*n* = 8; 4 pens with barrows and 4 pens with gilts) and 3 pigs were housed in a pen. Pigs were fed for 56 d until 123 kg BW based on 3 phases: grower 2 for 21 d (54 to 75 kg), finishing 1 for 17 d (75 to 100 kg) and finishing 2 for 18 d (100 to 123 kg). Treatments were CON (a typical corn-soybean meal-based diet with 18.0%, 13.8%, 12.7% CP for grower 2, finishing 1, and finishing 2; supplementing up to the third limiting AA); LCP (with 13.5%, 11.4%, 10.4% CP for grower 2, finishing 1, and finishing 2; supplementing up to the sixth AA); and LCPG (with 14.1%, 12.8%, 11.1% CP for grower 2, finishing 1, and finishing 2; LCP + Glu to match SID Glu within CON). All experimental diets were supplemented with crystalline AA to meet the AA requirements on NRC [32] and were formulated based on net energy (NE) with 2.6 Mcal/kg NE. The dietary composition is summarized in Table 3.

**Table 3:** Composition of diets (Exp. 3; as-fed basis)

Item	54 to 75 kg (Grower 2)			75 to 100 kg (Finishing 1)			100 to 120 kg (Finishing 2)		
	CON^a^	LCP^b^	LCPG^c^	CON^a^	LCP^b^	LCPG^c^	CON^a^	LCP^b^	LCPG^c^
Feedstuff, %									
Corn (yellow)	76.86	85.01	84.42	82.16	90.01	89.43	89.07	93.73	93.39
Soybean meal (48% CP)	18.80	10.90	10.90	14.40	6.78	6.78	7.56	3.00	3.00
L-Lys HCL	0.27	0.52	0.52	0.25	0.49	0.49	0.31	0.45	0.46
L-Met	0.03	0.10	0.10	0.00	0.07	0.07	0.00	0.04	0.04
L-Thr	0.06	0.17	0.17	0.06	0.16	0.16	0.09	0.15	0.15
L-Trp	0.00	0.04	0.05	0.01	0.05	0.05	0.02	0.05	0.05
L-Val	0.00	0.08	0.08	0.00	0.07	0.07	0.00	0.06	0.06
L-Ile	0.00	0.05	0.05	0.00	0.06	0.06	0.00	0.06	0.06
L-Glu	0.00	0.00	0.51	0.00	0.00	0.50	0.00	0.00	0.30
Dicalcium phosphate	0.90	1.03	1.04	0.77	0.90	0.90	0.69	0.77	0.77
Limestone	0.81	0.79	0.78	0.74	0.72	0.72	0.69	0.68	0.68
Vitamin premix^d^	0.03	0.03	0.03	0.03	0.03	0.03	0.03	0.03	0.03
Mineral premix^e^	0.15	0.15	0.15	0.15	0.15	0.15	0.15	0.15	0.15
Salt	0.22	0.22	0.22	0.22	0.22	0.22	0.22	0.22	0.22
Poultry fat	1.88	0.93	1.00	1.22	0.31	0.38	1.17	0.61	0.66
Calculated composition									
Dry matter, %	89.13	88.96	89.02	88.95	88.79	88.85	88.82	88.73	88.77
ME, kcal/kg	3,401	3,363	3,367	3,379	3,343	3,348	3,389	3,368	3,371
NE, kcal/kg	2,589	2,589	2,589	2,591	2,591	2,591	2,632	2,632	2,632
CP, %	15.62	13.01	13.27	13.93	11.41	11.66	11.33	9.85	10.00
SID^f^ Lys, %	0.85	0.85	0.85	0.73	0.73	0.73	0.61	0.61	0.61
SID Met + Cys, %	0.48	0.48	0.48	0.42	0.42	0.42	0.36	0.36	0.36
SID Thr, %	0.52	0.52	0.52	0.46	0.46	0.46	0.40	0.40	0.40
SID Trp, %	0.15	0.15	0.15	0.13	0.13	0.13	0.11	0.11	0.11
SID Val, %	0.60	0.55	0.55	0.54	0.48	0.48	0.42	0.41	0.41
SID Ile, %	0.53	0.45	0.45	0.46	0.39	0.39	0.35	0.33	0.33
SID Phe, %	0.65	0.51	0.51	0.58	0.44	0.44	0.45	0.37	0.37
SID His, %	0.38	0.30	0.30	0.34	0.27	0.26	0.27	0.23	0.23
SID Leu, %	1.24	1.06	1.05	1.14	0.97	0.96	0.98	0.88	0.88
SID Glu, %	2.38	1.89	2.38	2.12	1.63	2.12	1.68	1.39	1.68
Ca, %	0.59	0.59	0.59	0.52	0.52	0.52	0.46	0.46	0.46
STTD^g^ P, %	0.27	0.27	0.27	0.24	0.24	0.24	0.21	0.21	0.21
Analyzed composition									
CP, %	17.05	13.50	14.05	13.80	11.40	12.80	12.65	10.35	11.10

^a^Control diet (CON)

^b^Low CP diet (LCP)

^c^Low CP diet + L-Glu (LCPG)

^d^Vitamin premix: the vitamin premix provided the following per kilogram of complete diet: 6,613.8 IU of vitamin A as vitamin A acetate, 992.0 IU of vitamin D_3_, 19.8 IU of vitamin E, 2.64 mg of vitamin K as menadione sodium bisulfate, 0.03 mg of vitamin B_12_, 4.63 mg of riboflavin, 18.52 mg of D-pantothenic acid as calcium pantothenate, 24.96 mg of niacin, and 0.07 mg of biotin

^e^Mineral premix: the trace mineral premix provided the following per kilogram of complete diet: 4.0 mg of Mn as manganous oxide, 165 mg of Fe as ferrous sulfate, 165 mg of Zn as zinc sulfate, 16.5 mg of Cu as copper sulfate, 0.30 mg of I as ethylenediamine di-hydroiodide, and 0.30 mg of Se as sodium selenite

^f^*SID* Standardized ileal digestible

^g^*STTD* Standardized total tract digestible

### Growth performance and backfat thickness

Body weight and feed disappearance were recorded weekly to calculate average daily gain (ADG), average daily feed intake (ADFI), and gain to feed ratio (G:F) as indicators of growth performance. In Exp. 2 and 3, the backfat thickness was measured weekly using the ultrasound (Lean-Meater, RENCO, Golden Valley, MN, USA) at P2 (6.5 cm from the mid-dorsal line at the last rib) in all pigs.

### Sample collection

In Exp. 1, on d 28, 24 pigs (1 pig/pen, 8 pens/treatment) representing a median BW of each pen were euthanized by exsanguination after the penetration of a captive bolt to the head. Mucosa samples from the duodenum and jejunum were stored at –80 °C for further measurements of tumor necrosis factor-alpha (TNF-α), indicating the immune status, and protein carbonyls, indicating the oxidative stress status. Tissue samples from the duodenum and jejunum were stored at –80 °C for further measurements of tight junction proteins (Claudin-1, Occludin-1, and Zonula occludens-1), as indicators of intestinal barrier function. Tissue samples from the jejunum were used to analyze mRNA of AA transporters (CAT-1, b^0,+^AT, rBAT, y^+^LAT, 4F2hc, and B^0^AT) as indicator of AA absorption. Tissue samples from the duodenum and jejunum were stored in 10% buffered formalin at room temperature for histology evaluation, as indicator of intestinal epithelial health.

At the end of Exp. 2 and 3, blood samples were collected from 24 pigs (1 pig/pen, 8 pens/treatment) representing a median BW of each pen. Blood samples were collected from the jugular vein into 10-mL vacutainers with K2-EDTA (366643, BD, Franklin Lakes, NJ, USA) on ice and centrifuged (3,000 × *g* at 4 °C for 15 min) to obtain plasma. The plasma samples were divided into 5 aliquots and stored at –80 °C for further analysis of blood ureic nitrogen (BUN) and insulin.

In Exp. 3, at the end of the experiment, fecal samples were freshly collected into a sterile container and stored at –80 °C after snap freezing in liquid nitrogen to analyze the fecal microbiota by 16S rRNA sequencing.

### Immune and oxidative stress parameters

Mucosa samples were ground using a tissue grinder (Tissuemiser, Thermo Fisher Scientific Inc., Rockford, IL, USA) at 4 °C. The homogenate was centrifuged for 10 min at 14,000 × *g* at 4 °C. The obtained supernatant was split in 5 aliquots and stored at –80 °C to further quantify the concentrations of total protein, TNF-α, and protein carbonyls using a colorimetric method. The absorbance was read using a plate reader (Synergy HT, BioTek Instruments, Winooski, VT, USA) and the Gen5 Data Analysis Software (BioTek Instruments).

The total protein of mucosa samples was analyzed with Pierce BCA Protein Assay Kit (23227, Thermo Fisher Scientific Inc.), following the instructions of the manufacturer. The TNF-α concentrations in mucosa from duodenum and jejunum were analyzed using Porcine TNF-α Immunoassay ELISA Kit (PTA00, R&D System Inc., Minneapolis, MN, USA) as previously described by Xu et al. [33]. The detection limit range for TNF-α ELISA was 2.8 to 5.0 pg/mL. Concentrations of TNF-α in mucosa samples were expressed as ng/mg protein. The protein carbonyl concentrations in mucosa samples from the duodenum and jejunum were analyzed using Protein Carbonyl ELISA Kit (STA-310, Cell Biolabs, Inc., San Diego, CA, USA) as described by Weaver et al. [34]. Before the analysis, the samples were diluted to reach the concentration of total protein at 10 µg/mL. The detection limit range for protein carbonyls was 0 to 7.5 nmol/mg protein. Concentrations of protein carbonyls in mucosa samples were expressed as nmol/mg protein.

### Intestinal morphology

Tissue samples from duodenum and jejunum fixed in buffered formalin were transversely cut in two sections and placed in cassettes. The samples were sent to North Carolina State University Histology Laboratory (Raleigh, NC, USA) in 70% ethanol solution for dehydration, embedment, and staining according to their internal standard protocol. Staining was done using hematoxylin and eosin dyes. Villus height and crypt depth were measured under a camera (Infinity 2-2 digital CCD, Lumenera Corporation, Ottawa, CN) attached to a microscope (Olympus, Tokyo, Japan). Lengths of 10 well-oriented intact villi and their associated crypt were measured in each slide. Then, the ratio of villus height to crypt depth (VH:CD) was calculated. One person executed all the analyses of intestinal morphology as previously reported by Holanda and Kim [35] and Sun et al. [36].

### Tight junction proteins

The tight junction proteins were measured as previously reported by Duarte and Kim [37]. Briefly, the total protein of the duodenum and jejunum were extracted by using RIPA Lysis and Extraction Buffer (content: 25 mmol/L Tris-HCl, pH 7.6, 150 mmol/L NaCl, 1% NP-40, 1% sodium deoxycholate, 0.1% SDS) (89900, Thermo Fisher Scientific Inc.), with the supplementation of Halt Protease Inhibitor Cocktail (100 ×) (87786, Thermo Fisher Scientific Inc.). The total protein concentration was measured using the Pierce BCA Protein Assay Kit (23227, Thermo Fisher Scientific Inc.). In the next step, an individual sample was diluted to obtain 30 μg/mL of protein, then they were mixed with 4 × Laemmli Sample Buffer (161-0747, Bio-rad, Hercules, CA, USA) at the ratio of 4:1 in volume. The mixture was heated in a water bath at 95 °C for 5 min to get denatured protein before loading on SDS-PAGE gels. Samples were run in the SDS-PAGE gels accompanied by the Precision Plus Protein WesternC Standards (161-0376, Bio-rad). After gel running, proteins were electron transferred to Immun-Blot PVDF Membrane (162-0175, Bio-rad) and incubated with 5% nonfat dry milk overnight at 4 °C. The membrane was incubated with primary antibodies against Occludin-1 (ab31721, Abcam, Cambridge, MA, USA), ZO-1 (SC-10804, Santa Cruz, Paso Robles, CA, USA), Claudin-1 (ab129119, Abcam), and β-actin (SC-47778, Santa Cruz) at a dilution of 1:1,000 for 2 h at room temperature. Then after washing with Tween-20 diluted buffer, the membrane was incubated with a different corresponding secondary antibody according to the host of the primary antibody at the dilution of 1:5,000 for 45 min at room temperature (Goat Anti-Rabbit IgG H&L, ab6721, and Rabbit Anti-Mouse IgG H&L, ab6728, Abcam). Protein band densities were detected by DAB Substrate Kit (34002, Thermo Fisher Scientific Inc.) and quantified with AlphaImager 2200 (Alpha Innotech, San Leandro, CA, USA).

### Expression of amino acid transporters

The expression of amino acid transporters was measured as previously described by Jang et al. [38]. Total RNA of the jejunum was isolated from jejunal tissues by TRIzol Reagent (15596, Thermo Fisher Scientific Inc.). The RNA was synthesized to first-strand cDNA according to the instruction of SuperScript III First-Strand Synthesis System (18080-05, Life technology, Grand Island, NY, USA). Primers for amino acid transporters were designed using Oligo 7.0 (Table 4). Real-time PCR was performed using iQ5 Optical System (Bio-rad) with a total volume of 20 μL mixture containing 1 ng synthesized cDNA, appropriate primers, and SYBR Green Supermix (170-8882, Bio-rad). β-actin was used as the housekeeping gene to normalize relative gene expression. The PCR amplification was initiated at 95 °C for 3 min, followed by 40 cycles of 95 °C for 10 s, 59 °C for 30 s, and 72 °C for 30 s. Fold changes and relative gene expression levels were calculated using the comparative CT (2^−^^ΔΔCT^) method.

**Table 4:** Primers used for real-time PCR

Gene	Primer	Sequence (5'→3')	Size, bp	Accession number
*ASCT2*	Forward	GCCAGCAAGATTGTGGAGAT	206	DQ231578
	Reverse	GAGCTGGATGAGGTTCCAAA		
*B*^0^*AT1*	Forward	CACAACAACTGCGAGAAGGA	155	DQ231579
	Reverse	CCGTTGATAAGCGTCAGGAT		
*CAT-1*	Forward	TGCCCATACTTCCCGTCC	192	NM_001012613
	Reverse	GGTCCAGGTTACCGTCAG		
*b*^0,+^*AT*	Forward	ATCGGTCTGGCGTTTTAT	144	NM_001110171
	Reverse	GGATGTAGCACCCTGTCA		
*y*^+^*LAT1*	Forward	GCCCATTGTCACCATCATC	216	NM_001110421
	Reverse	GAGCCCACAAAGAAAAGC		
*4F2hc*	Forward	CTCGAACCCACCAAGGAC	174	XM_003361818
	Reverse	GAGGTGAGACGGCACAGAG		
*Pept-1*	Forward	CCCAGGCTTGCTACCCAC	144	NM_214347
	Reverse	ACCCGATGCACTTGACGA		
*rBAT*	Forward	TTTCCGCAATCCTGATGTTC	146	NM_001123042
	Reverse	GGGTCTTATTCACTTGGGTC		
β-actin	Forward	TGCGGGACATCAAGGAGAAG	216	XM_003357928
	Reverse	AGTTGAAGGTGGTCTCGTGG		

### Blood parameters

The concentrations of BUN and insulin were measured by colorimetric methods using commercially available kits according to the instructions of the manufacturers. The concentration of BUN was determined using the Urea Nitrogen (BUN) Colorimetric Detection Kit (EIABUN, Invitrogen, Carlsbad, CA, USA). Before the assay, the plasma samples were diluted (1:20) to reach the working range of 0.156 to 10 mg/dL. The absorbance was read at 450 nm and the concentration of BUN was expressed as mg/dL. The concentration of insulin was measured using the Insulin DuoSet ELISA Kit (DY8056-05, R&D System Inc.). The working range of the standard was 15.6 to 1,000 pmol/L. The absorbance was read at 450 with wavelength correction set to 540 nm and the concentration of insulin in plasma was expressed as pmol/L.

### Diversity and relative abundance of fecal microbiota

Fecal samples were used to extract total DNA content to analyze the microbiome. The DNA Stool Mini Kit (#51604, Qiagen; Germantown, MD, USA) was used to extract total DNA following the instructions of the manufacturer. The extracted DNA samples were sent to Mako Medical Laboratories (Raleigh, NC, USA) for microbiota sequencing using the 16S rRNA amplicon sequencing as previously reported by Duarte et al. [39]. The sequence data analysis, alignment to GreenGenes and MicroSeq databases, alpha and beta diversity plot generation, and operational taxonomic unit (OTU) table generation were performed by the Ion Reporter Software Suite (version 5.2.2) of bioinformatics analysis tools (Thermo Fisher Scientific Inc.). Samples were analyzed using Ion Reporter’s Metagenomics 16S workflow powered by Qiime (version w1.1). The OTU data were transformed to relative abundance before statistical analysis. The relative abundances of microbiota < 0.05% within each level were combined as “Others”.

### Statistical analysis

Pigs were allotted in a randomized complete block design using sex and the initial BW as blocks. The experimental unit was the pen for the growth performance and backfat thickness data. The selected pig with a median BW within a pen was considered the experimental unit for other measurements. The dietary treatments were the main effect and the initial BW and sex blocks were handled as random effects. Data were analyzed using the Mixed procedure in SAS version 9.4 (SAS Inc., Cary, NC, USA). Data from Exp. 3 were analyzed to assure there were no carryover effects from Exp. 2. The means were separated using the LSMEANS statement in SAS. When a treatment effect was significant or tended to be significant, a pairwise comparison was made using the PDIFF with the adjusted Tukey option in SAS. Statistical differences were considered significant with *P* < 0.05. Tendency was considered when 0.05 ≤ *P* < 0.10.

## Results

### Growth performance and backfat thickness

In Exp. 1, the LCP and LCPT diets increased (*P* < 0.05) the BW at d 7 and 14 compared with the CON diet (Table 5), whereas LCPT diet tended to increase (*P* = 0.099) the BW at d 29 compared with CON diet. The LCP and LCPT diets increased (*P* < 0.05) the ADG and G/F from d 0 to 7 compared with the CON diet. The LCP diet tended to increase (*P* = 0.075) the ADFI from d 0 to 7, whereas the LCPT diet increased (*P* < 0.05) ADFI from d 7 to 14 compared with the CON diet. However, overall, there was no difference among treatments on the growth performance parameters of pigs. In Exp. 2, there was no difference among treatments on the BW, ADG, and ADFI of pigs (Table 6). However, the VLCP diet tended to reduce (*P* = 0.071) the G/F from d 7 to 14 and reduced (*P* < 0.05) it in the overall experimental period. Additionally, LCP and VLCP diets increased (*P* < 0.05) the backfat thickness of pigs compared with the CON diet. In Exp. 3, there was no difference among treatments on the BW and ADFI of pigs during all experimental periods (Table 7). In grower 2, the LCPG diet tended to increase (*P* = 0.084) the ADG of pigs when compared with the CON diet. In finishing 1, the LCP diet reduced (*P* < 0.05) the ADG compared with the CON diet. However, there was no difference among treatments on the ADG of pigs in finishing 2 and overall period. The LCPG diet reduced (*P* < 0.05) the G/F of pigs in finishing 2 compared with CON, whereas there was no difference among treatments in G/F of pigs in the overall period. Additionally, the backfat thickness of finishing pigs did not differ among treatments during the experimental periods.

**Table 5:** Growth performance of pigs (20 to 40 kg) fed low CP diets with additional L-Trp supplementation (Exp. 1)

Item	CON^1^	LCP^2^	LCPT^3^	SEM	*P* value
BW, kg					
Initial	19.6	19.7	19.7	1.1	0.930
d 7	23.4^b^	24.3^a^	24.2^a^	1.3	0.005
d 14	28.0^b^	28.9^a^	29.2^a^	1.5	0.011
d 21	33.2^B^	34.0^AB^	34.3^A^	1.7	0.099
d 28	40.2	40.6	41.2	1.8	0.350
ADG, kg/d					
d 0 to 7	0.54^b^	0.66^a^	0.64^a^	0.04	0.003
d 7 to 14	0.65	0.66	0.71	0.04	0.149
d 14 to 21	0.75	0.74	0.74	0.04	0.828
d 21 to 28	0.99	0.94	0.98	0.04	0.480
d 0 to 28	0.73	0.75	0.77	0.03	0.393
ADFI, kg/d					
d 0 to 7	1.09^B^	1.21^A^	1.19^AB^	0.07	0.075
d 7 to 14	1.30^b^	1.37^ab^	1.44^a^	0.08	0.018
d 14 to 21	1.48	1.49	1.47	0.07	0.989
d 21 to 28	1.92	1.88	1.91	0.08	0.947
d 0 to 28	1.45	1.49	1.51	0.07	0.463
G:F					
d 0 to 7	0.50^b^	0.54^a^	0.55^a^	0.01	0.012
d 7 to 14	0.50	0.48	0.50	0.02	0.495
d 14 to 21	0.50	0.50	0.51	0.02	0.560
d 21 to 28	0.52	0.50	0.51	0.01	0.248
d 0 to 28	0.51	0.50	0.52	0.01	0.385

^1^Control diet (CON)

^2^Low CP diet (LCP)

^3^Low CP diet + L-Trp (LCPT)

^a,b^Means without a common superscript letter within a row differ (*P* < 0.05), *n* = 30

^A,B^Means without a common superscript letter within a row tend to differ (0.05 ≤ *P* < 0.10), *n* = 30

**Table 6:** Growth performance and backfat thickness of pigs fed diets with low CP formulation (Exp. 2)

Treatment	CON^1^	LCP^2^	VLCP^3^	SEM	*P* value
BW, kg					
Initial	34.2	34.2	34.3	4.2	0.988
d 7	39.6	40.2	39.9	4.7	0.715
d 14	47.5	49.6	48.5	4.8	0.866
d 21	53.8	55.0	54.1	5.3	0.872
ADG, kg/d					
d 0 to 7	0.78	0.85	0.81	0.08	0.363
d 7 to 14	1.06	1.10	1.04	0.08	0.687
d 14 to 21	0.97	1.02	0.99	0.11	0.819
d 0 to 21	0.94	0.99	0.94	0.06	0.588
ADFI, kg/d					
d 0 to 7	1.91	1.92	2.05	0.21	0.423
d 7 to 14	2.15	2.26	2.31	0.15	0.331
d 14 to 21	2.40	2.57	2.60	0.17	0.278
d 0 to 21	2.15	2.25	2.32	0.17	0.320
G/F					
d 0 to 7	0.41	0.44	0.40	0.02	0.169
d 7 to 14	0.50^A^	0.49^AB^	0.45^B^	0.03	0.071
d 14 to 21	0.40	0.40	0.38	0.04	0.672
d 0 to 21	0.43^a^	0.44^a^	0.41^b^	0.02	0.015
Backfat thickness, mm					
d 21	5.08^b^	6.04^a^	6.29^a^	0.64	0.021

^1^Control diet (CON)

^2^Low CP diet (LCP)

^3^Very low CP diet (VLCP)

^a,b^Means without a common superscript letter within a row differ (*P* < 0.05), *n* = 24

^A,B^Means without a common superscript letter within a row tend to differ (0.05 ≤ *P* < 0.10), *n* = 24

**Table 7:** Growth performance and backfat thickness of finishing pigs fed low CP diets with net energy and additional L-Glu supplementation (Exp. 3)

Treatment	CON^1^	LCP^2^	LCPG^3^	SEM	*P* value
BW, kg					
Initial	54.2	54.1	54.1	6.0	0.999
d 21	79.0	79.7	80.7	6.3	0.788
d 38	100.2	98.3	101.0	7.2	0.612
d 56	123.6	122.0	123.2	7.9	0.872
ADG, kg/d					
d 0 to 21	1.19^B^	1.22^AB^	1.26^A^	0.03	0.084
d 21 to 38	1.24^a^	1.09^b^	1.20^ab^	0.06	0.030
d 38 to 56	1.30	1.32	1.23	0.08	0.115
d 0 to 56	1.24	1.21	1.23	0.04	0.662
ADFI, kg/d					
d 0 to 21	2.92	2.95	3.04	0.16	0.478
d 21 to 38	3.47	3.34	3.37	0.20	0.678
d 38 to 56	3.50	3.67	3.54	0.21	0.355
d 0 to 56	3.27	3.30	3.30	0.18	0.954
G:F					
d 0 to 21	0.41	0.41	0.42	0.02	0.539
d 21 to 38	0.36	0.33	0.36	0.01	0.118
d 38 to 56	0.37^a^	0.36^ab^	0.35^b^	0.01	0.037
d 0 to 56	0.38	0.37	0.37	0.01	0.360
Backfat thickness, mm					
d 0	5.87	5.67	5.64	0.69	0.835
d 21	8.79	9.35	9.13	0.46	0.661
d 38	11.00	10.35	11.27	0.52	0.461
d 55	12.96	13.10	12.96	0.92	0.984

^1^Control diet (CON)

^2^Low CP diet (LCP)

^3^Low CP diet + L-Glu (LCPG)

^a,b^Means without a common superscript letter within a row differ (*P* < 0.05), *n* = 24

^A,B^Means without a common superscript letter within a row tend to differ (0.05 ≤ *P* < 0.10), *n* = 24

### Intestinal morphology, immune, and oxidative stress status

In Exp. 1, LCP and LCPT diets increased (*P* < 0.05) villus height in the duodenum compared with the NC diet (Table 8). In addition, the LCP diet also increased (*P* < 0.05) villus height and crypt depth compared to the CON diet. LCPT increased (*P* < 0.05) villus height and crypt depth compared to CON and LCP. LCPT diet tended to increase (*P* = 0.088) the VH:CD of pigs when compared with CON. However, villus width in both duodenum and jejunum were not affected by the treatments.

**Table 8:** Intestinal morphology, immune and oxidative stress status of growing pigs fed low CP diets with additional L-Trp supplementation (Exp. 1)

Treatment	CON^1^	LCP^2^	LCPT^3^	SEM	*P* value
Duodenum					
Villus height, µm	480^b^	491^a^	492^a^	2	0.004
Villus width, µm	169	170	172	1	0.352
Crypt depth, µm	371	373	374	3	0.324
VH:CD ratio^4^	1.29	1.31	1.32	0.01	0.107
TNF-α, pg/mg of protein	11.36^B^	9.82^A^	9.54^A^	0.52	0.072
Protein carbonyl, nmol/mg of protein	3.87	3.82	3.54	0.16	0.488
Jejunum					
Villus height, µm	368^c^	372^b^	380^a^	1	< 0.001
Villus width, µm	120	123	123	1	0.273
Crypt depth, µm	252^b^	256^a^	257^a^	1	0.008
VH:CD ratio^4^	1.46^AB^	1.45^B^	1.48^A^	0.01	0.088
TNF-α, pg/mg protein	7.60^B^	6.42^A^	5.79^A^	0.71	0.063
Protein carbonyl, nmol/mg of protein	3.95	3.92	3.69	0.18	0.664

^1^Control diet (CON)

^2^Low CP diet (LCP)

^3^Low CP diet + L-Trp (LCPT)

^4^Villus height to crypt depth ratio

^a,b^Means without a common superscript letter within a row differ (*P* < 0.05), *n* = 30

^A,B^Means without a common superscript letter within a row tend to differ (0.05 ≤ *P* < 0.10), *n* = 30

In Exp. 1, LCP and LCPT diets tended to decrease the concentration of TNF-α in both duodenum (*P* = 0.072) and jejunum (*P* = 0.063) when compared with the CON diet (Table 8). However, the concentration of protein carbonyls in the duodenum and jejunum was not affected by dietary treatments.

### Expression of AA transporters in jejunum

**Fig. 1:**
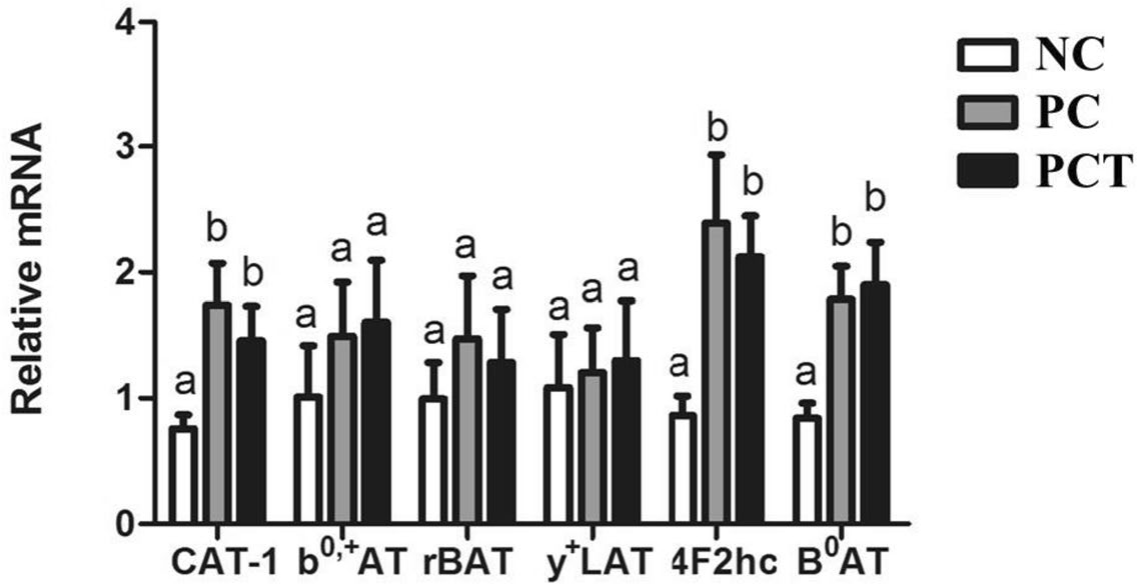
Expression of mRNA related to amino acid transporters in jejunum of growing pigs fed low CP diets with additional L-Trp supplementation (Exp. 1). ^a,b^Means without a common letter differ (*P* < 0.05)

In Exp. 1, LCP and LCPT diets increased (*P* < 0.05) the concentrations of mRNA of CAT-1, 4F2hc, and B^0^AT when compared with the CON diet (Fig. 1). Whereas the concentrations of mRNA of b^0,+^AT, rBAT, and y^+^LAT did not differ among treatments.

### Tight junction proteins

**Fig. 2:**
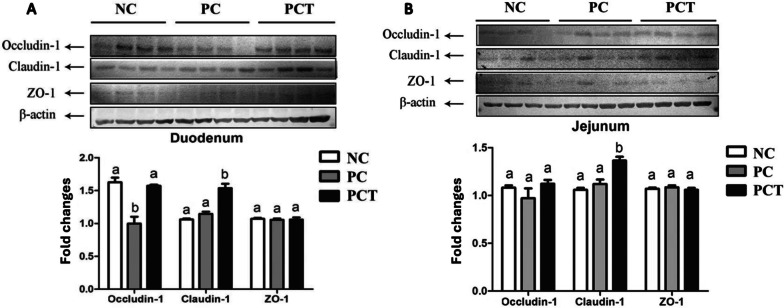
Concentration of tight junction proteins in duodenum (**A**) and jejunum (**B**) of growing pigs fed low CP diets with additional L-Trp supplementation (Exp. 1). ^a,b^Means without a common letter differ (*P* < 0.05)

In Exp. 1, the LCP diet reduced (*P* < 0.05) the concentration of Occludin-1 in the duodenum of pigs compared with CON and LCPT diets (Fig. 2A). Whereas the LCPT diet increased (*P* < 0.05) the concentration of Claudin-1 in the duodenum of pigs when compared with CON and LCP diets. In the jejunum, the LCPT diet increased (*P* < 0.05) the concentration of Claudin-1 when compared with CON and LCP diets (Fig. 2B).

### Blood parameters

In Exp. 2, the VLCP diet reduced (*P* < 0.05) the concentration of BUN in pigs when compared with CON and LCP diets (Table 9). In Exp. 3, the LCPG diet tended to reduce the concentrations of BUN (*P* = 0.087) and increase the concentration of insulin (*P* = 0.093) in pigs when compared with the CON diet (Table 10).

**Table 9:** Blood parameters of pigs (34 to 54 kg) fed diets with low CP formulation (Exp. 2)

Treatment	CON^1^	LCP^2^	VLCP^3^	SEM	*P* value
BUN^4^, mg/dL	7.57^a^	8.57^a^	3.28^b^	0.75	< 0.001
Insulin, pmol/L	21.36	11.28	24.17	6.99	0.341

^1^Control diet (CON)

^2^Low CP diet (LCP)

^3^Very low CP diet (VLCP)

^4^*BUN* Blood urea nitrogen

^a,b^Means without a common superscript letter within a row differ (*P* < 0.05), *n* = 24

**Table 10:** Blood parameters of finishing pigs fed low CP diets with net energy and additional L-Glu supplementation (Exp. 3)

Treatment	CON^1^	LCP^2^	LCPG^3^	SEM	*P* value
BUN^4^, mg/dL	6.05^A^	4.52^AB^	4.03^B^	0.60	0.087
Insulin, pmol/L	11.57^A^	14.29^AB^	22.15^B^	4.15	0.093

^1^Control diet (CON)

^2^Low CP diet (LCP)

^3^Low CP diet + L-Glu (LCPG)

^4^BUN = blood urea nitrogen

^A,B^Means without a common superscript letter within a row tend to differ (0.05 ≤ *P* < 0.10), *n* = 24

### Relative abundance and diversity of the fecal microbiota

In Exp. 3, the relative abundance of fecal microbiota did not differ among treatments at the phylum level (Table 11). At the family level, the LCP and LCPG diets reduced (*P* < 0.05) the relative abundance of Streptococcaceae in the feces of pigs when compared with the CON diet, whereas the LCP diet reduced (*P* < 0.05) the abundance of Erysipelotrichaceae in feces of pigs compared with CON diet. At the species level, the LCP and LCPG diets reduced (*P* < 0.05) the relative abundance of *Streptococcus alactolyticus* in the feces of pigs when compared with the CON diet (Table 11). Whereas the LCPG diet tended to increase (*P* = 0.077) the relative abundance of *Clostridium butyricum* in the feces of pigs when compared with the CON diet. The alpha diversity of fecal microbiota at the family level did not differ among treatments (Table 12). Whereas at the species level, the LCP diet tended to reduce (*P* = 0.071) the alpha diversity of fecal microbiota in pigs estimated with Chao1 richness estimator when compared with the CON diet.

**Table 11:** Relative abundance of fecal microbiota in finishing pigs fed low CP diets with net energy and additional L-Glu supplementation (Exp. 3)

Treatment	CON^1^	LCP^2^	LCPG^3^	SEM	*P* value
Phylum					
Bacteroidetes	43.24	39.14	38.72	2.60	0.350
Firmicutes	31.33	28.31	31.79	2.54	0.452
Proteobacteria	18.14	24.99	22.53	3.05	0.298
Spirochaetes	4.75	5.36	5.56	1.84	0.923
Tenericutes	1.43	1.15	0.79	0.31	0.369
Others	1.11	1.05	0.61	0.35	0.354
Family					
Prevotellaceae	30.73	29.28	28.21	3.31	0.861
Succinivibrionaceae	13.55	21.82	18.96	3.27	0.219
Veillonellaceae	4.79	6.94	6.61	1.01	0.292
Porphyromonadaceae	6.16	5.28	5.96	1.00	0.810
Lachnospiraceae	4.53	5.48	6.00	1.14	0.226
Spirochaetaceae	4.67	5.29	5.52	1.86	0.915
Ruminococcaceae	4.48	3.99	4.88	0.87	0.770
Streptococcaceae	6.11^a^	2.02^b^	2.26^b^	1.16	0.002
Eubacteriaceae	2.03	1.99	2.05	0.27	0.985
Bacteroidaceae	2.16	1.82	1.77	0.44	0.784
Erysipelotrichaceae	1.83^a^	0.88^b^	1.23^ab^	0.24	0.037
Lactobacillaceae	0.87	0.94	1.24	0.21	0.413
Acidaminococcaceae	1.03	0.89	0.97	0.31	0.867
Anaeroplasmataceae	1.15	0.89	0.55	0.32	0.416
Rikenellaceae	0.86	0.89	0.76	0.39	0.971
Sphingobacteriaceae	1.03	0.53	0.67	0.50	0.742
Cytophagaceae	0.80	0.66	0.44	0.18	0.409
Flavobacteriaceae	0.80	0.38	0.61	0.20	0.334
Others	8.83	6.38	6.43	1.32	0.346
Species					
*Prevotella copri*	28.15	29.46	31.28	5.91	0.918
*Streptococcus alactolyticus*	17.86^a^	8.13^b^	7.06^b^	3.73	0.020
*Prevotella stercorea*	9.63	10.44	10.51	2.13	0.948
*Succinivibrio dextrinosolvens*	8.60	9.93	8.60	3.58	0.911
*Faecalibacterium prausnitzii*	5.65	5.82	5.60	1.49	0.992
*Prevotella* sp.	3.53	5.51	3.43	2.51	0.459
*Treponema porcinum*	2.17	4.18	4.88	1.93	0.596
*Treponema Treponema*	3.70	2.45	2.58	1.97	0.831
*Roseburia faecis*	1.39	2.75	2.71	1.23	0.278
*Clostridium butyricum*	1.56^B^	1.93^AB^	2.94^A^	0.70	0.077
*Lactobacillus ruminis*	1.64	1.59	2.31	0.65	0.505
*Clostridium* sp.	1.44	1.03	2.46	0.51	0.147
*Butyrivibrio crossotus*	0.05	1.41	1.28	1.03	0.595
*Streptococcus infantarius*	0.93	0.64	0.63	0.28	0.631
*Roseburia intestinalis*	0.37	0.98	0.79	0.51	0.220
*Clostridium sacchar*	0.56	0.62	0.89	0.26	0.314
*Selenomonas* sp.	0.67	0.62	0.54	0.25	0.925
*Desulfovibrio D168*	0.66	0.85	0.31	0.32	0.506
*Campylobacter coli*	0.67	0.62	0.30	0.30	0.496
*Selenomonas lipol*	0.20	0.72	0.65	0.19	0.152
*Ruminococcus gnavus*	0.35	0.66	0.52	0.16	0.240
Others	10.21	9.66	10.07	1.94	0.960

^1^Control diet (CON)

^2^Low CP diet (LCP)

^3^Low CP diet + L-Glu (LCPG)

^a,b^Means without a common superscript letter within a row differ (*P* < 0.05), *n* = 24

^A,B^Means without a common superscript letter within a row tend to differ (0.05 ≤ *P* < 0.10), *n* = 24

**Table 12:** Alpha diversity of fecal microbiota in finishing pigs fed low CP diets with net energy and additional L-Glu supplementation (Exp. 3)

Treatment	CON^1^	LCP^2^	LCPG^3^	SEM	*P* value
Family					
Chao1	78.24	63.75	70.38	6.85	0.346
Shannon	3.73	3.43	3.58	0.20	0.582
Simpson	0.85	0.83	0.84	0.02	0.717
Species					
Chao1	56.36^A^	43.63^B^	49.13^AB^	3.68	0.071
Shannon	3.45	3.47	3.54	0.25	0.916
Simpson	0.82	0.83	0.82	0.04	0.982

^1^Control diet (CON)

^2^Low CP diet (LCP)

^3^Low CP diet + L-CP (LCPG)

^A,B^Means without a common superscript letter within a row tend to differ (0.05 ≤ *P* < 0.10), *n* = 24

## Discussion

Supplemental crystalline AA is essential for pig production to reduce the use of protein supplements, maintaining the ideal protein profile in the diets [3, 6, 40]. A diet with a deficient AA supply reduces the growth performance, whereas an excess of AA is costly and negatively impacts the environment by increasing nitrogen excretion [41]. In the current study, a reduction of 2% to 3% with supplemental Lys, Met, Thr, Trp, Val, and Ile did not affect the overall growth performance, regardless of the feeding phase. Additionally, lowering CP levels enhanced intestinal health by increasing the expression of AA transporters and tight junction proteins, consequently improving the epithelial layer as seen in the increased villus height in the small intestine of growing-finishing. Additional Trp and Glu supplementation in low CP diets improved intestinal health without affecting growth. However, a further reduction of 4% in dietary CP with supplemental Lys, Met, Thr, Trp, Val, Ile, Phe, and His impaired feed efficiency, which can be associated with the increased NE intake, considering that the diets in Exp. 2 were formulated using the ME formulation. The feed efficiency and BFT did not differ in Exp. 3 when the diets were formulated using the NE formulation.

It is important to state that three experiments were conducted to address the hypothesis of this study and the interpretation of the results also accounted with the different phase feeding among experiments. In Exp. 1, reducing the CP from 18% to 16%, supplementing Lys, Met, Thr, Trp, and Val with ME formulation in diets of pigs from 20 to 40 kg BW, enhanced the intestinal health and the expression of AA transporters, whereas without affecting the growth performance of growing pigs in overall period. Rocha et al. [8] reported that dietary CP can be reduced up to 16.1% or 16.3% without compromising the ADG and feed efficiency of growing pigs, respectively. However, during the first two weeks of Exp. 1, the growth performance was improved when pigs were fed diets with a low CP diet. These results indicated that the benefits of a low CP diet on growth performance could be observed in pigs up to 30 kg BW.

Additional Trp supplementation to a low CP diet, increasing the SID Trp level from 0.17% to 0.22% further enhanced intestinal health by increasing the villus height and the expression of tight junction proteins on the intestinal mucosa. In the past years, a greater SID Trp to Lys ratio, exceeding the NRC [32] recommendations, has been suggested [42, 43]. Shen et al. [14] previously reported that Trp supplementation increased the growth performance by increasing hypothalamic 5-HT production and reducing the concentration of stress hormones, however, in Exp. 1, the additional 0.05% SID Trp did not affect the growth performance of pigs. Tryptophan has also been reported to enhance the intestinal functions in pigs by regulating the expression of the mammalian target of rapamycin (mTOR) [18, 44, 45], which can explain the improvement observed in the intestinal mucosa in Exp. 1. According to Qin et al. [46], the mTOR plays an important role in the intestinal barrier functions. The mTOR signaling pathway increases the proliferation of intestinal epithelial cells [18, 47] and, reduces apoptosis [48], consequently sustaining the villus height. Furthermore, in Exp. 1 the Trp supplementation increased the abundance of Claudin-1 in the ileum and jejunum of pigs. Wang et al. [18] reported that the increased concentration of Trp in the epithelial cells due to the increased expression of AA transporters activates the mTOR pathways enhancing the protein synthesis, increasing the synthesis of Claudin-4, Occludin, ZO-1, and ZO-2 in intestinal epithelial cells. However, in Exp. 1, the expressions of AA transporters were not further increased with the additional supplementation of Trp.

In Exp. 1, a low CP diet supplementing Lys, Met, Thr, Trp, and Val decreased the abundance of Occludin-1 in the duodenum. However, an additional 0.05% Trp over the NRC [32] requirement recovered the expression of Occludin-1 in the duodenum to the level in the control diet and improved the expression of Claudin-1 in the duodenum and jejunum. Together, these results indicate that the Trp level in a low CP diet meeting the NRC [32] requirements may not be sufficient for the synthesis of tight junction proteins, whereas further Trp supplementation can recover or increase the expression of tight junction proteins. Moreover, in low CP diets, NEAA, peptides, and isoflavones removed with the reduction of soybean meal could, speculatively, impair other biological functions [8]. Studies have reported that deficiency or excess of Trp negatively affects the expression of tight junction proteins in fish [49] and pigs [45]. Tight junction proteins are multifunctional protein complexes present between adjacent epithelial cells [50] representing an important part of the intestinal barrier function. Tight junction proteins prevent paracellular diffusion of antigens across the epithelium [51]. Therefore, AA levels in the diet would have an important role in regulating the expression of tight junction regulators [18, 45].

Besides the role of barrier functions, the small intestine plays a vital role in AA absorption, and it is susceptible to changes in AA transporters by dietary interventions [52]. Following digestion, the released AA are absorbed mainly in the proximal jejunum by neutral (B^0^AT1, and ASCT2), cationic (rBAT/b^0,+^AT, CAT-1, and 4F2hc/y^+^LAT1) or anionic AA transporters (EAAT2, and EAAT3) [53]. Studies have demonstrated that dietary AA is involved in AA transporter regulation [54, 55]. In Exp. 1, the low CP diet supplementing Lys, Met, Thr, Trp, and Val increased the expression of CAT-1, 4F2hc, and B^0^AT. Lysine, a cationic AA, is transported by CAT-1. Methionine and threonine, neutral AA, are transported by B^0^AT. Moreover, 4F2hc is an important component of the y^+^ system and participates in Na^+^-independent transport of large neutral amino acids (LNAA) including Trp, Tyr, and the branched-chain amino acids (BCAA) [56, 57]. According to Li et al. [1], low CP diets play a role in the regulatory mechanism of AA transporters affecting the AA pools. Considering that all crystalline AA supplemented in the diets are promptly available for absorption, supplemental AA requires more transporters in the small intestine. According to Bröer and Fairweather [58], the AA level would stimulate the expression of transporters in the mammalian intestine, similar to the findings reported in the current study. However, the additional Trp in a low CP diet did not further enhance the expression of AA transporters.

In Exp. 2, reducing the CP from 16.7% to 14.8% by supplementing Lys, Thr, Trp, Met, Val, and Ile with ME formulation in the diet of pigs from 34 to 54 kg BW increased the BFT, whereas without affecting the growth performance. A concern in reducing the CP is the increase in the NE content within the diets [7, 30]. The increase in the backfat thickness observed in Exp. 2 may be associated with the increase in NE intake that increased by 316 kcal/d when the CP was reduced from 16.7% to 14.8%. However further increment of 237 kcal/d on the NE intake did not further increase the backfat thickness. These results are in agreement with Acosta et al. [31] who reported that an increment of 260 kcal/d NE increased the backfat thickness of finishing pigs. The reduction in protein supplements in the diet decreases the metabolic cost for the digestion of proteins, increasing the NE content in the diet [3, 7]. According to Kerr et al. [59], when a low CP diet is supplemented with AA it reduces the heat increment, consequently decreasing the energy requirement for maintenance. Furthermore, when the CP was further reduced from 16.7% to 12.6% by supplementing Lys, Thr, Trp, Met, Val, Ile, Phe, and His, the feed efficiency and the BUN were decreased.

The excess protein intake would be metabolized to increase the concentration of urea in the blood [60]. Therefore, reducing the nitrogen intake decreased the BUN in Exp. 2 and Exp. 3. However, in Exp. 2, the BUN decreased when the dietary CP was reduced from 16.7% to 12.6% with supplementation of eight AA (Lys, Met, Thr, Trp, Val, Ile, Phe, and His), whereas it was not affected when the CP was reduced from 16.7% to 14.8% with supplementation of six AA (Lys, Met, Thr, Trp, Val, and Ile). Conversely, in Exp. 3, the BUN tended to decrease in a low CP diet supplementing Lys, Met, Thr, Trp, Val, Ile, and Glu. Blood urea nitrogen has been correlated with urinary nitrogen excretion [61] and hence can be used to predict nitrogen excretion [62]. Following the model suggested by Kohn et al. [62] the urinary nitrogen excretion was reduced from 12.6 to 5.6 g/d in Exp. 2 and from 23.5 to 16.9 g/d in Exp. 3, by reducing the CP in the diets.

The dietary energy level can also affect the nitrogen utilization affecting the BUN. According to Cai et al. [63], in a deficient energy diet, AA would be catabolized to be used to supply the energy needs. However, in this study, all diets met the energy requirements, whereas the ratio of NE and nitrogen intake was greater in Exp. 3 (164 kcal/g nitrogen) than in Exp. 2 (111 kcal/g nitrogen) even with the diets in Exp. 2 formulated using the ME system. This indicates that energy intake should be considered to improve nitrogen utilization. When the NE system was used in Exp. 3, reducing the CP from 15.6% to 13.0%, 13.9% to 11.4%, and 11.3% to 9.9%, for grower 2, finisher 1 and 2, respectively, with supplemental Lys, Met, Thr, Trp, Val, and Ile, the growth performance, and BFT were not affected. Rocha et al. [8] recently conducted a meta-analysis and concluded that reducing dietary CP up to 11.6% and 11.4% do not compromise the daily gain and feed efficiency of pigs, considering the 2 finishing phases. In addition, considering energy and other nutrients are not limiting factors, the reduction of nitrogen excretion would reduce the negative environmental impact, enhancing the sustainability of production.

The abundance of potentially harmful bacteria in feces was reduced, as expected, in low CP diets. It has been established that dietary protein level, protein source, and the AA profile are associated with the microbiota composition and consequently with its interaction with the host [64–66]. The level of CP and the AA availability in the diets can directly affect microbiota [67]. By altering the CP level in the diet, the AA profile of that non-supplemented AA would be affected altering the nitrogen substrate for microbial growth. In Exp. 3, the reduction of CP, supplementing up to the sixth AA affected the ratio of SID Val, Ile, Leu, Phe, His, and the NEAA to Lys in the diet compared with the control diet. Considering that the crystalline AA is promptly absorbed in the small intestine, lesser undigested proteins would reach the large intestine in low CP diets. Consequently, lower dietary CP first affects the proliferation of protein-degrading bacteria [3]. In Exp. 3, a low CP diet reduced the abundance of Erysipelotrichaceae and Streptococcaceae. The reduction in the abundance of Streptococcaceae was mainly attributed due to the increased abundance of *Streptococcus alactolyticus*. *Streptococcus* spp. is one of the major bacteria fermenting proteins in the intestine of monogastric animals [68, 69]. Chen et al. [70] reported that reducing 3% of dietary CP modulated the intestinal microbiota by decreasing the abundance of *Streptococcus* and increasing *Lactobacillus* and *Bifidobacterium*, in ileal digesta of growing pigs. However, in Exp. 3, the alpha diversity was decreased in the feces of pigs fed low CP diets, whereas it was restored when Glu was supplemented. This result suggests that a minimum nitrogen amount is necessary for the proliferation of intestinal microbiota [10, 71].

In the LCPG treatment, the SID Glu was matched with the level in the CON diet. Interestingly, the supplemental Glu tended to increase the abundance of *Clostridium butyricum*. *C. butyricum* has been used as a probiotic for promoting health benefits to the host [70, 72–74]. It has been reported that Glu can alter the composition of the microbiota and increase diversity [75]. The trends to increase the microbial diversity and reduced BUN, in conjuction, may indicate a betterment of the efficiency of nitrogen utilization and a shift in the nitrogen excretion from urine to feces in the form of bacterial protein. The CP level also affects the fermentation kinetic. Wen et al. [76] reported that the CP level in the diet affected the microbial metabolites in the large intestine by altering the microbiota composition. According to Zervas and Zijlstra [77], the shift in the nitrogen excretion from urine to feces reduces ammonia emission. It is important to mention that the shift on microbiota composition should be toward a more health-benefiting microbiota.

In Exp. 2 and 3, the feeds had 73% to 93% of corn during growing to finishing, which would provide a high content of glucose for the pigs. However, the level of insulin in pigs during Exp. 2 and 3 were not different between CON and Low CP diets, even with the blood collected in different phases. In Exp. 3, Glu supplementation increased the concentration of insulin in the blood of pigs fed a low CP diet, which may indicate an enhancement in glucose metabolism. The role of Glu in regulating insulin secretion is dependent on glucose concentration [78, 79]. According to Stanley [25], besides controlling the nitrogen balance, Glu regulates the secretion of insulin by Glu dehydrogenase (GDH). High glucose levels inhibit the oxidation of Glu increasing the synthesis of glutamine. Consequently, the released glutamine increases the secretion of insulin [24, 25]. Intriguingly, the LCPG diet was formulated to contain the same amount of SID Glu as the control diet. The difference in the insulin levels may be attributed to the faster absorption of crystalline Glu compared with protein bound Glu, whereas further investigation is warranted to investigate deeper the differences on the functional roles of Glu in different forms.

High insulin level is correlated with increased lipogenesis [80]. Conversely, according to Taylor and Halperin [81], glutamate down-regulates lipogenesis in white adipose tissue of rats by inhibiting the activity of pyruvate dehydrogenase and consequently reducing fat deposition. According to Hu et al. [29], supplemental Glu can reduce fat deposition in subcutaneous adipose tissue, while increasing the deposition of intramuscular fat in pigs. Doran et al. [82] reported that enzymes related to lipogenesis are more expressed in muscle than in subcutaneous adipose tissue in pigs fed low CP diets. Therefore, considering that the supplemental Glu increased the insulin in plasma without affecting the backfat thickness observed in Exp. 3, it can be suggested that the supplemental Glu to low CP diet may have increased the intramuscular fat of finishing pigs. Further research is warranted to investigate carcass traits and meat quality by evaluating the expression of genes associated with fat deposition in different formulations of low CP diets.

## Conclusion

Reducing dietary CP from 18% to 16% with ME formulation and supplementation of Lys, Met, Thr, Trp, and Val enhanced the intestinal health and the expression of AA transporters in the small intestine of pigs from 20 to 40 kg. Supplementation of 0.05% Trp above the requirement seems to have a functional effect by improving intestinal integrity whereas maintaining growth performance. For pigs from 34 to 54 kg, reducing CP from 17.7% to 15.0% with Lys, Thr, Trp, Met, Val, Ile, and constant ME increased BFT without affecting growth performance. However, further reducing CP from 17.7% to 12.8% with Lys, Thr, Trp, Met, Val, Ile, Phe, and His impaired growth performance by reducing feed efficiency. In pigs from 54 to 120 kg, reducing CP with Lys, Thr, Trp, Met, Val, Ile, and constant NE reduced potentially harmful bacteria without affecting growth performance and BFT. Additionally, Glu supplementation to a low CP diet further enhanced N utilization, glucose metabolism, and fecal microbiota composition, indicating benefits to intestinal health and the environmental sustainability. Overall, reducing CP by 2% to 3% in corn-soybean meal-based diet fed to growing-finishing pigs can be achieved without compromising growth performance. This is possible through supplementation with Lys, Met, Thr, Trp, Val, and Ile, along with maintaining NE to prevent excessive energy intake and fat deposition. Further supplementation with Trp and Glu in low CP diets could improve intestinal health and enhance nitrogen and glucose metabolism. Consequently, these nutritional strategies offer the potential for more economical, efficient, and sustainable practices within the pig production. This could possibly benefit both producers and the environment by reducing feed costs, minimizing nitrogen excretion, and potentially improving animal welfare and health.

## Data Availability

All data generated or analyzed during this study are available from the corresponding author upon reasonable request.

## Abbreviations

AA: Amino acid
ADFI: Average daily feed intake
ADG: Average daily gain
BFT: Backfat thickness
BW: Body weight
BUN: Blood urea nitrogen
CP: Crude protein
GF: Gain to feed ratio
MDA: Malondialdehyde
ME: Metabolizable energy
NE: Net energy
NEAA: Non-essential amino acid
SID: Standardized ileal digestible
TNF-α: Tumor necrosis alpha
VHCD: Villus height to crypt depth ratio
